# Two-Dimensional Echocardiography in the Assessment of Long-Term Prognosis in Patients with Pulmonary Arterial Hypertension

**DOI:** 10.1371/journal.pone.0114443

**Published:** 2014-12-08

**Authors:** Ling-yue Sun, Hang Zhao, Yu Kang, Xue-dong Shen, Zong-ye Cai, Jie-yan Shen, Ben He, Cheng-de Yang

**Affiliations:** 1 Department of Cardiology, Ren Ji Hospital, School of Medicine, Shanghai Jiao Tong University, Shanghai, China; 2 Department of Rheumatology, Ren Ji Hospital, School of Medicine, Shanghai Jiao Tong University, Shanghai, China; Nippon Medical School Graduate School of Medicine, Japan

## Abstract

**Objective:**

To investigate the relationship between cardiac diastolic dysfunction and outcomes in patients with pulmonary arterial hypertension (PAH) and to clarify the potential effect of two-dimensional echocardiography (2D-echo) on prognostic value in patients with PAH.

**Methods:**

Patients diagnosed with PAH (as WSPH (World Symposia on Pulmonary Hypertension) classification I) confirmed by right heart catheterization (RHC), received targeted monotherapy or combination therapy. 2D-echo parameters, World Health Organization (WHO) functional classification and 6-minute walking distance (6MWD) were recorded. The clinical prognosis of patients was assessed by the correlation between echo parameters and clinical 6MWD using receiver operating characteristic (ROC) curve analysis.

**Results:**

Fifty-eight patients were included. Left and right ventricular diastolic dysfunction (LVDD and RVDD) scores measured by 2D-echo had good correlation with 6MWD at baseline (r_LVDD_  = −0.699; r_RVDD_  = −0.818, both P<0.001) and at last follow-up (r_LVDD_  = −0.701; r_RVDD_  = −0.666, both P<0.001). Furthermore, bi-ventricular (LVDD+RVDD) scores measured by 2D-echo had a better correlation with 6MWD at baseline and last follow-up (r = −0.831; r = −0.771, both P<0.001). ROC curve analysis showed that the area under curves (AUCs) for LVDD score, RVDD score and (LVDD+RVDD) scores were 0.823 (P<0.0001), 0.737 (P = 0.0002), and 0.825 (P<0.0001), respectively. Compared with ROC analysis of other single parameters, cardiac diastolic function score was more accurate in predicting survival in patients with PAH.

**Conclusion:**

LVDD score, RVDD score and (LVDD+RVDD) scores yielded a comprehensive quantitative assessment of LV and RV diastolic function that correlated moderately with clinical functional parameters and might be useful in the assessment of PAH.

## Introduction

Echocardiography (echo) is used mainly for preliminary screening in patients with suspected pulmonary arterial hypertension (PAH). In the absence of right ventricular (RV) outflow tract obstruction, systolic pulmonary artery pressure (SPAP) is estimated from the RV systolic pressure (RVSP; calculated by addition of the tricuspid regurgitation pressure gradient to the estimated right atrial pressure). Recently, echo parameters have been used to evaluate RV function, by tricuspid annular plane systolic excursion (TAPSE) [1]–[3], Tei index [4]–[6], mitral valve peak E velocity [7], mitral valve peak A velocity, and E/A [8]. Compared with cardiac magnetic resonance imaging (MRI), which is the gold standard for cardiac functional detection [9], [10], few echo parameters predicting the prognosis of PAH have been identified. However, due to high costs and the complexity of its use, cardiac MRI is limited in routine clinical examination [9]. As a result of its non-invasive nature, low cost, and lack of radiation, echo is a widely available imaging technique that is particularly suitable for tentative clinical diagnosis and follow-up studies of PAH. Thus, it is of clinical importance to investigate echo parameters in evaluating heart function and predicting the prognosis of patients with PAH. The aim of this study was to identify valuable echo parameters in order to comprehensively assess the clinical prognosis of patients with PAH.

## Materials and Methods

### Patients

Between January 2008 and December 2011, we prospectively studied 58 consecutive patients aged 18 to 78 years. All patients underwent clinical evaluation, including hematological examination (NT-proBNP, D-dimer, arterial blood gas analysis, liver function test, rheumatologic blood test), electrocardiogram (ECG), chest radiography, pulmonary function test, high resolution CT (HR-CT) scan, and 6MWD on the same day that they also underwent echo evaluation. RHC was performed within 48 hours of echo examination. Inclusion criteria were mean pulmonary arterial pressure (MPAP) >25 mm Hg, pulmonary arterial wedge pressure (PAWP) ≤15 mm Hg, and pulmonary vascular resistance (PVR)>3 Wood units (under RHC). Diagnosis of PAH was based on criteria from the 5^th^ WSPH - patients were required to have confirmed WSPH classification I. Exclusion criteria were WSPH- I with atrial fibrillation and other irregular rhythm, WSPH-II, III, IV, and V pulmonary hypertension (PH), or non-PH. Regarding 2D echo, the image data obtained from the patients with rhythm abnormalities would not be available and accurate. According to 5^th^-WSPH [11], 28 patients (48.3%) were diagnosed with connective tissue disease (CTD), 22 patients (37.9%) with idiopathic PAH, 7 patients (12.1%) with congenital heart disease (4 with atrial septal defects [1 after repair], 2 with ventricular septal defects, and 1 with patent ductus arteriosus), and 1 patient (1.7%) with portal hypertension. Twenty-eight patients (48.3%) were classified as WHO functional class III–IV, with the remaining 30 patients (51.7%) being WHO functional class II. All patients provided written informed consent. The study protocol was approved by the Institutional Review Board of Shanghai Jiao Tong University and the Ethics Committee of Ren Ji Hospital.

### Instruments and Methods

Echo imaging was performed in all study participants in the left decubitus or supine position using a GE vivid E9 equipped with a M5S transducer (frequency 1.8–3.6 MHz; GE Healthcare, Milwaukee, WI). In the first instance, two-dimensional (2D) and Doppler images were acquired using the MS5 transducer. To ensure inclusion of the entire left and right ventricles within the pyramidal scan volume, all echo datasets were acquired using the wide-angled full-volume mode over three consecutive cardiac cycles during a single breath-hold. Three datasets were obtained for each patient. Echo recordings and measurements were performed according to the American College of Cardiology Foundation (ACCF)/American Society of Echocardiography (ASE) appropriate use criteria [12]. The procedures were performed separately and independently by two cardiologists who were unaware of the assignment of the study.

### 2D-echo

**Figure 1:**
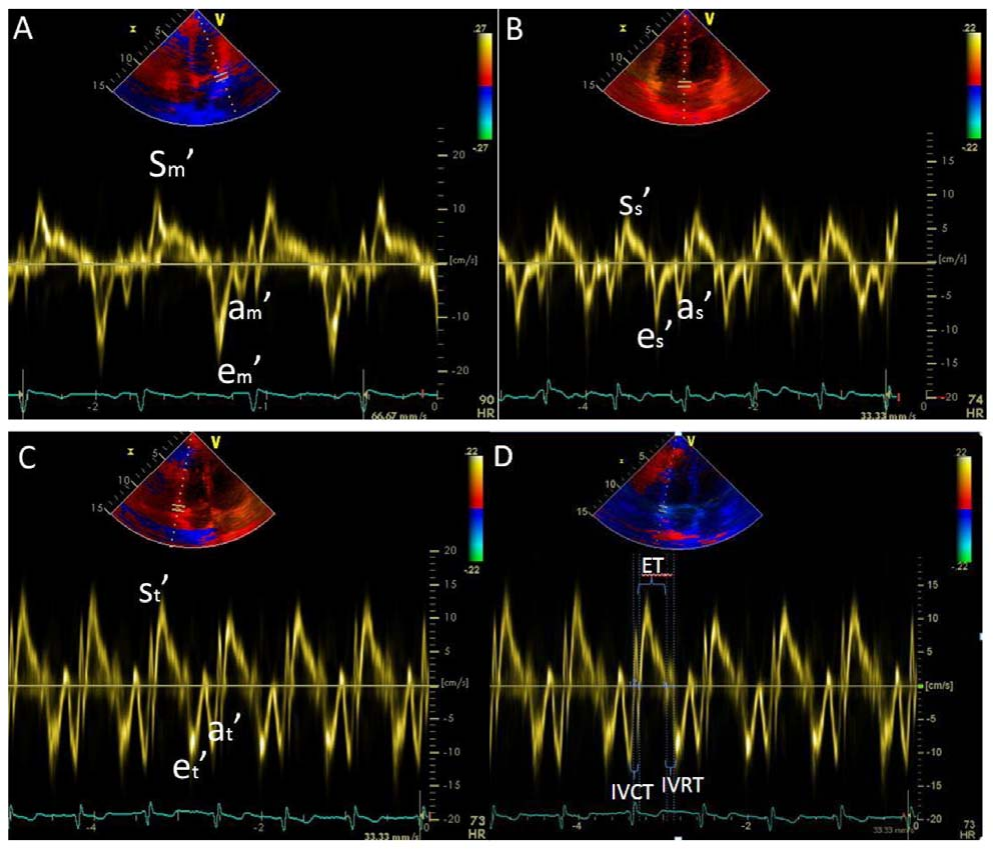
Tissue Doppler at the lateral and septal walls of the mitral annulus (A, B); Tissue Doppler at the lateral wall of the Tricuspid annulus (C); Tissue Doppler of Tei Index (D).

Standard views from the parasternal long and short axis and apical four-chamber views were used [6]. Flow velocities were obtained by using pulsed and continuous wave Doppler techniques, as proposed by the ASE [13], [14]. Mitral inflow velocities were obtained by pulsed-wave (PW) Doppler at the level of the mitral leaflet tips using color flow imaging for optimal alignment of the Doppler beam at the apical 4-chamber view. Measurements of mitral inflow included the mitral valve peak E velocity (E_m_), mitral valve peak A velocity (A_m_), the E_m_/A_m_ ratio and deceleration time (DT_m_) of mitral valve peak E. PW tissue Doppler imaging (DTI) was performed to acquire mitral annular velocities by setting the sample point at the lateral and septal sides of the mitral annulus. Measurements included the mitral annular lateral peak systolic velocity (S_m_′), mitral annular lateral e velocity (e_m_′), lateral a velocity (a_m_′); mitral annular septal systolic velocity (S_s_′), septal e velocity (e_s_′) and septal a velocity (a_s_′) (Fig. 1, A and B). Additionally, measurements of tricuspid inflow, including the tricuspid valve peak E velocity (E_t_), tricuspid valve peak A velocity (A_t_), the E_t_/A_t_ ratio, and deceleration time (DT_t_) of tricuspid valve peak E velocity, were recorded by PW Doppler at the level of tricuspid leaflet tips with the help of color flow imaging for optimal alignment of the Doppler beam. DTI of tricuspid annular velocity measurements included tricuspid annular peak systolic velocity (S_t_′), tricuspid annular lateral e velocity (e_t_′) and lateral a velocity (a_t_′) (Fig. 1, C). TAPSE was measured by the systolic anterior movement of the lateral annular of tricuspid valve under M-mode motion. RV end-diastolic area (RVEDA) and end-systolic area (RVESA) were assessed using manual planimetry, and RV fractional area change (FAC) was derived as follows: FAC  =  [(RVEDA -RVESA)/RVEDA]*100%. RV Tei index was determined as the sum of the isovolumic contraction time (IVCT) and isovolumic relaxation time (IVRT) divided by the ejection time (ET): Tei index  =  [(IVCT+IVRT)/ET] [15] (Fig. 1, D). RVSP was estimated by adding the right atrial pressure (RAP) to the transtricuspid pressure gradient (TPG), which was calculated from the peak TR velocity using the modified Bernoulli equation: TPG (mm Hg)  =  4V^2^. RAP was estimated to be 3, 8 or 15 mm Hg by visualizing the inferior vena cava (IVC) diameter and its collapse during inspiration under long-axis subcostal views [16]. RVSP was approximated to be SPAP in the absence of RV outflow obstruction. The RV outflow tract time-velocity integral was obtained by placing a 1-mm to 2-mm pulsed-wave Doppler sample volume in the proximal RV outflow tract just within the pulmonary valve when imaged from the parasternal short-axis view. PVR was calculated using the following equation: PVR (Wood units)  = 10 * TR velocity/RV outflow tract time-velocity integral +0.16 [17].

Measurement of cardiac morphology and function indicators included: left atrial (LA) diameter, left ventricular (LV) internal diameter, left atrium volume index (LAVI), LV ejection fraction (LVEF), right atrial (RA) diameter, RV internal diameter, RV end-diastolic volume (RVEDV), RV end-systolic volume (RVESV), RV stroke volume (RVSV), and RV ejection fraction (RVEF). According to ASE guidelines, the varying severity of LV diastolic dysfunction is reflected by all six conventional measures of LV filling, including e_m_′, e_s_′, E_m_/A_m_, E_m_/e_m_′, DT_m_, LAVI. LV diastolic function was divided into: normal, mild or grade I (impaired relaxation pattern), moderate or grade II (pseudo-normalization) and severe or grade III (restrictive filling) by integrated analysis of multiple LV diastolic parameters [18]. In order to analyze LV diastolic function intuitively and statistically, we quantified the grading scheme (normal, grade I, grade II and grade III) into LV diastolic function scores 1, 2, 3 and 4. According to the ASE guidelines [16], grading of RV diastolic dysfunction should be performed using echo measures, including E_t_/A_t_, E_t_/e_t_′ and DTt. Similar to LV diastolic function, RV diastolic function was graded and assigned a score as follows: normal (score  = 1), grade I (score  = 2; impaired relaxation), grade II (score  = 3; pseudo-normal filling), grade III (score  = 4; restrictive filling).

### 6-MWD and Borg dyspnea scale

The 6MWD Test was conducted according to American Thoracic Society guidelines [19]. Specifically, the test was performed using a long, flat, straight, enclosed corridor of at least 30 meters in length. SpO_2_ was monitored continuously with a wireless pulse oximeter. The following data were recorded: total distance walked (expressed in meters), SpO_2_, blood pressure and heart rate before and after the test. The Borg dyspnea scale was assessed in conjunction with the 6MWD Test.

### Treatment

All patients were treated with targeted therapies including endothelin receptor antagonists (ERA), phosphodiesterase type-5 inhibitors (5-PDEI), and prostacyclins, in addition to conventional drugs according to current guidelines [20]. Dopamine and levosimendan were given additionally to rescue acute right heart failure. One or two PAH-targeted combination therapies were added on to stable but unsatisfactory patients.

### Follow-up

All patients were followed-up via an outpatient clinic or hospitalization every 6 months ±2 weeks. The deadline of the last visit of the last patient was 30 June 2013. Patients underwent echo, 6MWD Test, as well as WHO functional classification and Borg dyspnea scale evaluation during every follow-up. Major adverse events, including death, lung transplantation, and heart-lung transplantation, were recorded.

### Statistical analysis

Normally distributed continuous variables were presented as mean ± SD. The Student's t test was used to compare values of clinical and echo parameters between baseline and the last follow-up. The degree of agreement between two modalities was assessed using the methods of Pearson's correlation coefficient (SPSS Statistics ver. 20.0, IBM Corporation, Armonk, NY, USA) [21], [22]. Cut-off values of the evaluated parameters which enabled the prediction of adverse events with the highest sensitivity and specificity were identified by means of receiver operating characteristic (ROC) curve analysis. These values were then used to predict the clinical prognosis of patients with PAH (Med Calc Software for Windows ver. 12.7, Acacialaan, Ostend, Belgium).. A P-value of less than 0.05 was considered to be statistically significant.

## Results

### Baseline

Demographic and clinical characteristics of all patients enrolled in this study are shown in Table 1 and Table 2. Fifty-eight patients were included in this study. The majority of patients (87.9%) were female, and the mean age was 42.9±15.9 years. Twenty-eight patients (48.3%) were diagnosed with CTD-PAH. Twenty-eight patients (48.3%) were classified as WHO functional class III–IV. The mean 6MWD (all patients) was 396.3±95.1 m. Hemodynamic parameters measured by RHC were: SPAP 73.0±23.6 mm Hg, DPAP 39.0±13.0 mm Hg, MPAP 51.7±15.4 mm Hg, PVR 11.6±5.3 Wood units, and CO 4.0±1.3 L/min.

**Table 1:** Baseline demographic and clinical characteristics in all patients.

Characteristics	
Age (years)	42.9±15.9
Female (%)	51 (87.9%)
Etiology (%)	
Connective tissue disease	28 (48.3%)
Idiopathic pulmonary arterial hypertension	22 (37.9%)
Congenital heart disease	7 (12.1%)
Portal hypertension	1 (1.7%)
Treatment	
Bosentan (monotherapy)	8
Ambrisentan (monotherapy)	1
Sildenafil (monotherapy)	7
Vardenafil (monotherapy)	9
Tadalafil (monotherapy)	7
Beraprost (monotherapy)	5
Iloprost (INH) (monotherapy)	1
Bosentan+ sildenafil or vardenafil or tadalafil	7
Vardenafil +bosentan or ambrisentan or beraprost	7
Beraprost + sildenafil or bosentan	6
Measurements of right heart catheterization	
Systolic pulmonary arterial pressure (mm Hg)	73.0±23.6
Diastolic pulmonary arterial pressure (mm Hg)	39.0±13.0
Mean pulmonary arterial pressure (mm Hg)	51.7±15.4
Pulmonary vascular resistance (Wood units)	11.6±5.3
Cardiac output (L/min)	4.0±1.3

Cardiac output: Cardiac output in CHD-PAH was effective pulmonary blood flow obtained by Fick's principle.

**Table 2:** Clinical and echocardiographic parameters in all patients at baseline and the last follow-up.

	Characteristics		*P* value
	Baseline	Last follow-up	
WHO			
I	0	6 (10.4%)	NS
II	30 (51.7%)	24 (41.4%)	NS
III	23 (39.7%)	14 (24.1%)	NS
IV	5 (8.6%)	14 (24.1%)	NS
Borg dyspnea scale	3.9±1.6	3.6±1.4	0.038
6MWD (m)	396.3±95.1	412.7±109.9	0.11
NT-proBNP (pg/ml)	653.8±229.1	509.5±173.4	0.351
Echocardiographic parameters (2-dimensional)			
Left ventricular			
Em (cm/s)	63.4±22.1	75.0±19.4	<0.001
Am (cm/s)	55.0±15.5	57.8±19.2	0.282
Em/Am	1.29±0.66	1.43±0.57	0.085
DTm (ms)	188±42	168±43	0.004
em′ (cm/s)	8.7±3.1	8.6±3.1	0.853
Em/em′	8.3±4.8	9.7±4.3	0.011
am′ (cm/s)	10.9±3.5	10.3±2.9	0.442
Sm′ (cm/s)	10.0±3.5	9.0±2.6	0.004
LAVI (ml/m2)	37.3±4.9	38.2±4.5	0.042
LVEF (%)	63.4±4.1	61.3±3.9	0.562
Septal es′ (cm/s)	8.0±2.7	7.3±2.6	0.104
Septal as′ (cm/s)	8.9±2.5	8.9±2.7	0.496
Septal Ss′ (cm/s)	8.5±2.4	7.5±1.7	0.007
Right ventricular			
Et (cm/s)	61.2±17.6	60.3±18.3	0.592
At (cm/s)	54.9±13.4	52.0±14.1	0.037
Et/At	1.22±0.57	1.30±0.64	0.22
St′ (cm/s)	9.6±2.9	9.2±3.5	0.125
et′ (cm/s)	10.3±3.3	10.6±3.7	0.072
Et/et(	6.5±2.6	6.3±2.7	0.385
at( (cm/s)	14.6±5.2	13.5±4.9	0.351
DTt (ms)	137±32	127±33	0.001
RAA (cm2)	22.1±9.8	20.9±9.0	0.034
TAPSE (mm)	15.7±3.1	15.5±3.3	0.641
Tei index	0.58±0.07	0.59±0.07	0.325
FAC (%)	0.34±0.06	0.32±0.05	0.026

6MWD: 6-minute walking distance; E_m_: Mitral valve peak E velocity; A_m_: Mitral valve peak A velocity; DT_m_: Deceleration time of mitral valve peak E; e_m_′/a_m_′: Lateral e/a velocity of mitral annulus by DTI; S_m_′: Mitral annular peak systolic velocity; LAVI: left atrium volume index; LVEF: left ventricular ejection fraction; E_t_: Tricuspid valve peak E velocity; A_t_: Tricuspid valve peak A velocity; S_t_′: Tricuspid annular peak systolic velocity; e_t_′/a_t_′: Lateral e/a velocity of tricuspid annulus by DTI; DT_t_: Deceleration time of tricuspid valve peak E; RAA: Right atrial area; TAPSE: tricuspid annular plane systolic excursion; FAC: Fractional area change.

As presented in Table 1, 19 patients received bosentan, 2 ambrisentan, 14 sildenafil, 17 vardenafil, 9 tadalafil, 14 beraprost, and 1 iloprost. Of all 58 patients, 38 patients received monotherapy. Over one-third of all patients (n = 20) received two or three add-on combination therapies during the long-term follow-up period, when clinical worsening occurred.

### Follow-up data

The mean duration of follow-up for all 58 patients was 28.3±16.1 months. Seventeen patients died and 1 underwent lung transplantation during a mean total duration of 28.3±16.1 (range 6 to 70) months of follow-up. The main adverse events occurred at an average of 20.9±11.7 months in 18 patients. The mean follow-up duration for the other 40 patients with PAH was 31.7±16.8 months. All data, including WHO functional class, Borg dyspnea scale, 6MWD, and parameters of 2D-echo of all 58 patients were recorded at the last follow-up (Table 2).

### Left ventricular diastolic function

**Figure 2:**
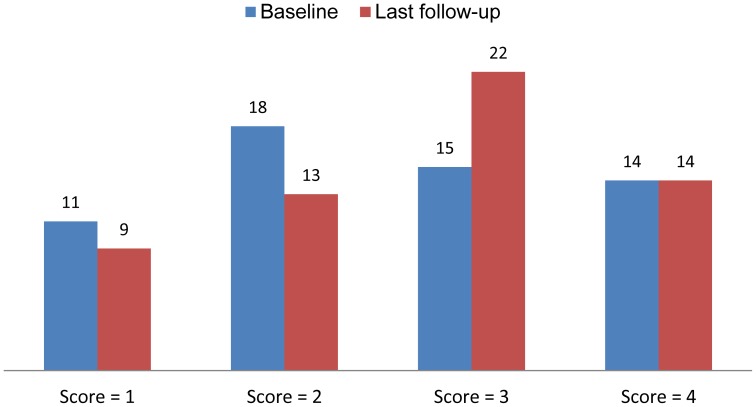
The number changes of patients in each score of LVDD at baseline and last follow-up.

**Figure 3:**
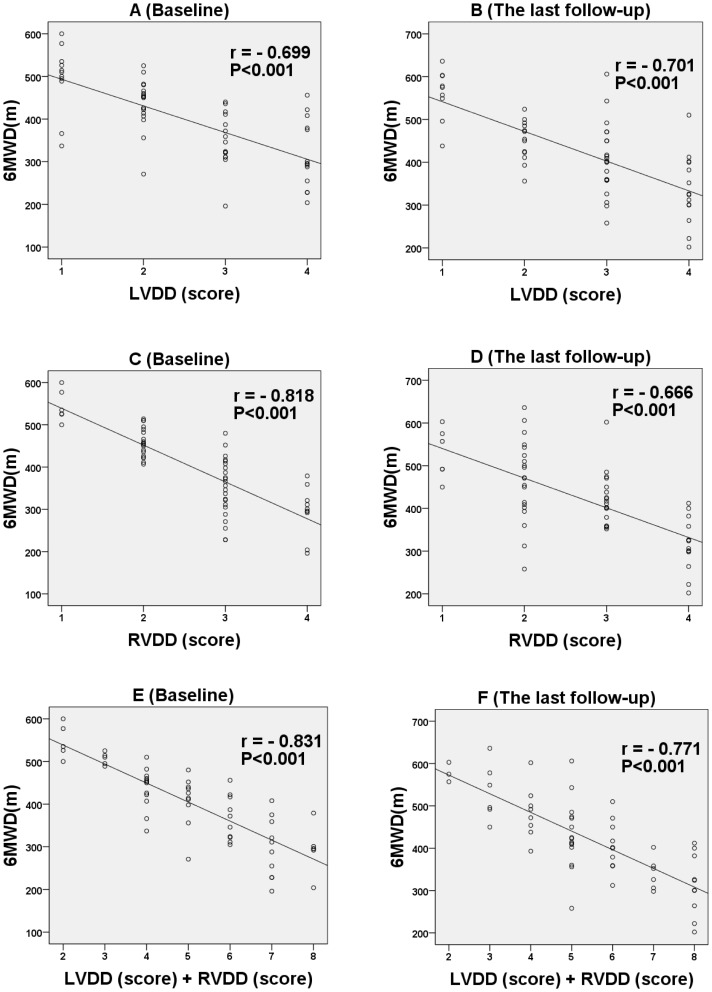
The relationship between LVDD score, RVDD score, (LVDD + RVDD) score and 6MWD at baseline (A, C, E) and the last follow-up (B, D, F).

LV diastolic grading scores and other diastolic parameters collected at baseline and last follow-up are listed in Table 3 and Fig. 2. Bivariate correlation analysis revealed a significant relationship between LV diastolic function score and 6MWD, with coefficient of correlations r_LVDD_  = −0.699 (P<0.001) at baseline and r_LVDD_  = −0.701 (P<0.001) at last follow-up, respectively (Fig. 3, A and B).

**Table 3:** Left ventricular diastolic dysfunction (LVDD) at baseline and the last follow-up.

	Normal	Mild (I)	Moderate (II)	Severe (III)
	Score = 1	Score = 2	Score = 3	Score = 4
	Baseline Last follow-up	Baseline Last follow-up	Baseline Last follow-up	Baseline Last follow-up
E_m_ (cm/s)	68.2±11.6 83.7±21.6	44.2±13.1 55.1±18.5	55.1±12.3 75.1±12.8	93.0±9.8 87.9±12.2
A_m_ (cm/s)	50.8±14.5 67.9±16.1	62.1±12.5 71.7±18.9	59.0±18.7 56.1±17.1	44.7±9.7 41.1±9.4
E_m_/A_m_	1.42±0.40 1.27±0.31	0.74±0.25 0.78±0.22	1.03±0.46 1.41±0.29	2.15±0.41 2.19±0.34
DT_m_ (ms)	198±21 157±41	236±28 223±20	168±11 160±29	141±17 136±28
e_m_′ (cm/s)	11.5±3.8 13.9±3.4	9.5±2.6 8.2±1.7	7.4±2.4 8.1±1.6	6.9±2.0 6.4±1.6
E_m_/e_m_′	6.5±2.5 6.3±1.9	4.8±1.2 6.9±2.2	7.8±2.1 9.7±3.1	14.7±5.0 14.5±4.2
S_m_′ (cm/s)	11.5±3.7 10.7±1.9	11.0±2.7 9.4±2.1	9.4±3.1 9.5±2.7	8.3±4.0 6.8±2.0
Septal es( (cm/s)	12.6±2.0 12.4±2.4	7.4±1.3 6.5±0.7	6.7±1.3 6.0±0.9	6.5±1.1 7.0±1.2
LAVI (mL/m2)	30.2±1.8 31.3±2.3	36.3±1.6 36.8±3.1	38.4±3.5 39.4±2.8	42.8±3.2 42.2±3.4

E_m_: Mitral valve peak E velocity; A_m_: Mitral valve peak A velocity; DT_m_: Deceleration time of mitral valve peak E; e_m_′: Lateral e velocity of mitral annulus by DTI; S_m_′: Mitral annular peak systolic velocity; LAVI: left atrium volume index.

### Right ventricular diastolic function

**Figure 4:**
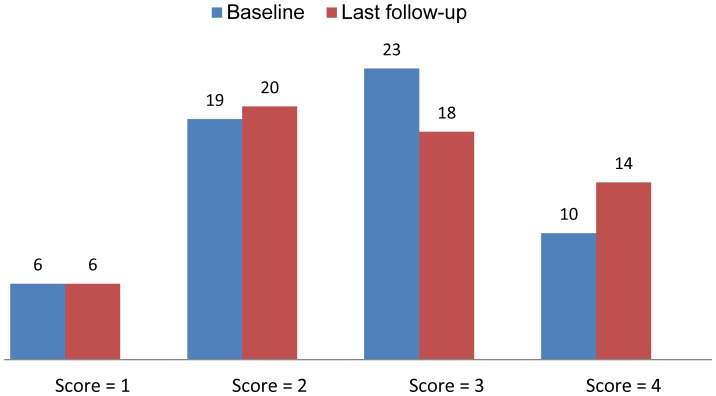
The number changes of patients in each score of RVDD at baseline and last follow-up.

RV diastolic scores and other echo parameters collected at baseline and last follow-up are listed in Table 4 and Fig. 4. The correlations of RV diastolic function score at baseline (r_RVDD_  = −0.818, P<0.001) and last follow-up (r_RVDD_  = −0.666, P<0.001) with 6MWD were significant (Fig. 3, C and D).

**Table 4:** Right ventricular diastolic dysfunction (RVDD) at baseline and the last follow-up.

	Normal	Mild(I)	Moderate(II)	Severe(III)
	Score = 1	Score = 2	Score = 3	Score = 4
	Baseline Last follow-up	Baseline Last follow-up	Baseline Last follow-up	Baseline Last follow-up
E_t_ (cm/s)	54.3±6.6 58.8±7.4	45.7±6.4 42.6±7.6	64.5±13.3 63.3±13.4	87.0±10.6 82.3±10.7
A_t_ (cm/s)	45.8±3.7 47.2±9.3	65.3±10.3 64.1±11.4	55.6±11.6 52.3±10.1	39.1±5.1 36.4±3.7
E_t_/A_t_	1.19±0.18 1.28±0.24	0.71±0.1 0.68±0.12	1.21±0.36 1.24±0.32	2.22±0.06 2.26±0.13
DT_t_ (ms)	157±22 154±29	141±37 127±41	141±30 131±33	109±9 111±10
e_t_′ (cm/s)	15.7±4.1 13.8±3.1	11.1±3.1 11.4±4.8	8.7±2.0 9.5±2.4	9.3±1.9 9.5±2.2
E_t_/e_t_′	3.7±1.2 4.4±0.9	4.4±1.3 4.5±2.4	7.5±1.1 6.8±1.2	9.7±2.4 9.1±2.4

E_t_: Tricuspid valve peak E velocity; A_t_: Tricuspid valve peak A velocity; e_t_′: Lateral e velocity of tricuspid annulus by DTI; DTt: Deceleration time of tricuspid valve peak E.

### Bi-ventricular diastolic function

To evaluate the whole cardiac diastolic function, we developed a new quantitative index. By adding the LV diastolic function score to the RV diastolic function score, total diastolic function scores were obtained to assess the entire cardiac diastolic function in patients with PAH. We observed that the total scores for bi-ventricular diastolic function correlated better with 6MWD than isolated LV or RV diastolic function scores, with coefficients of correlation r_LVDD+RVDD_  = −0.831 (P<0.001) at baseline and r_LVDD+RVDD_  = −0.771 (P<0.001) at last follow-up, respectively (Fig. 3, E and F).

### Other right ventricular echocardiographic parameters

**Figure 5:**
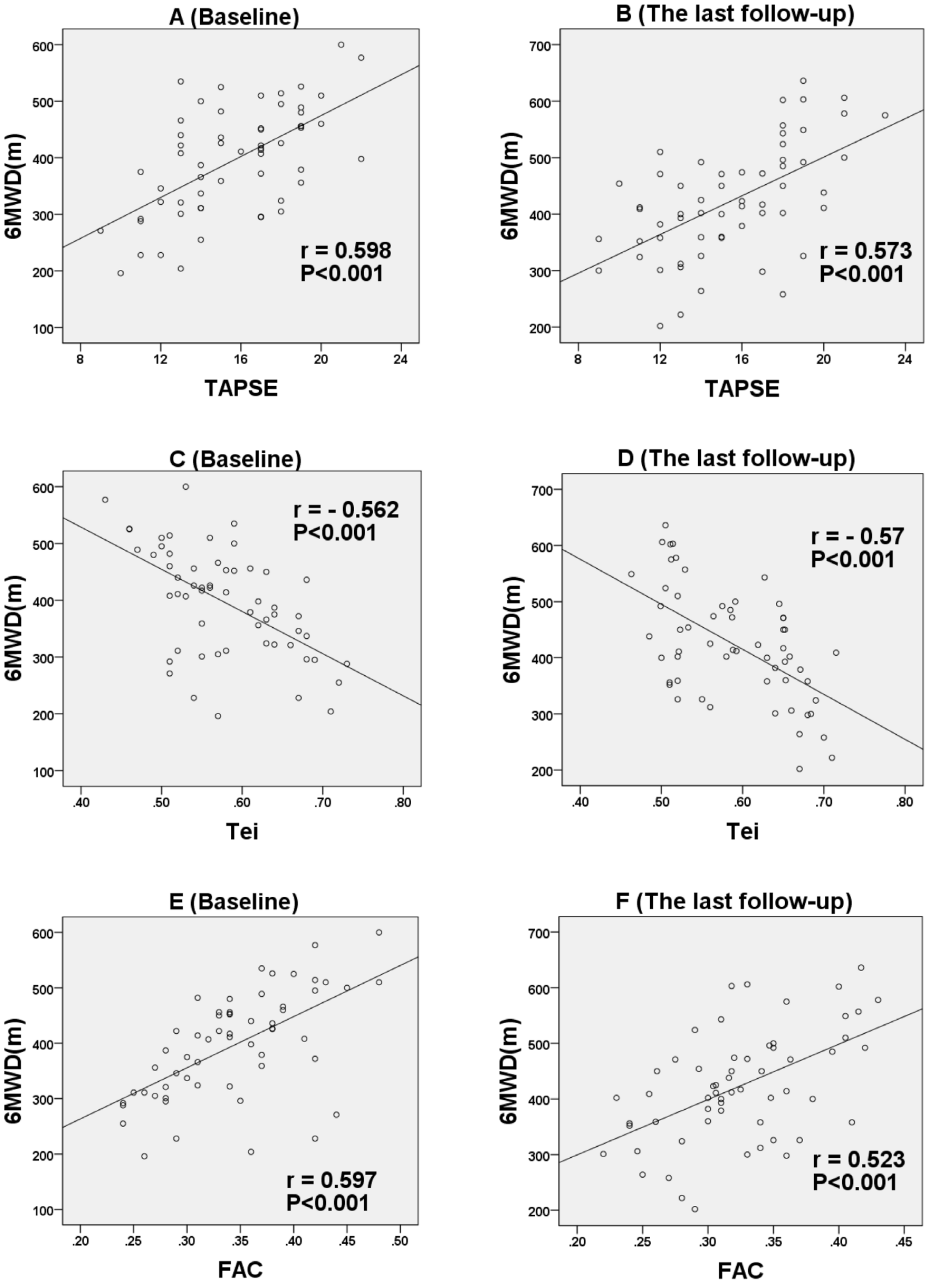
The relationship between TAPSE, Tei index, FAC and 6MWD at baseline (A, C, E) and the last follow-up (B, D, F).

Other RV echo parameters were measured to evaluate RV function by 2D-echo. Compared with quantitative indices of LV/RV/total diastolic function, the correlation between TAPSE (baseline: r = 0.598, P<0.001; last follow-up: r = 0.573, P<0.001, respectively), RV Tei index (baseline: r = −0.562, P<0.001; last follow-up: r = −0.57, P<0.001, respectively), FAC (baseline: r = 0.597, P<0.001; last follow-up: r = 0.523, P<0.001, respectively) with 6MWD were statistically less significant (Fig. 5).

### Clinical survival analysis

**Figure 6:**
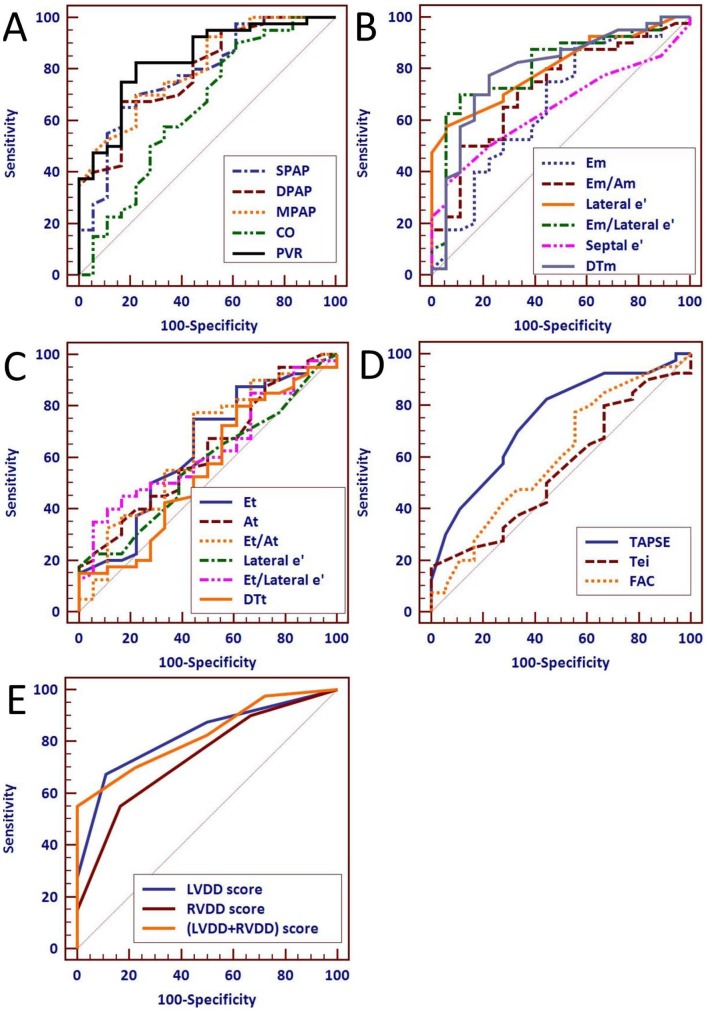
ROC curves for different parameters of RHC (A), different echocardiographic parameters of LV function (B) and RV function (C, D). ROC curves for LVDD score, RVDD score and (LVDD + RVDD) score (E).

Data for the area under the ROC curve for hemodynamic parameters of RHC (SPAP, DPAP, MPAP, CO and PVR) evaluating the clinical prognosis of patients with PAH were: AUC_SPAP_  = 0.780, P<0.0001; AUC_DPAP_  = 0.790, P<0.0001; AUC_MPAP_  = 0.807, P<0.0001; AUC_CO_  = 0.649, P = 0.0812; AUC_PVR_  = 0.838, P<0.0001, respectively (Table 5). Among these, PVR measured by RHC was more valuable and more accurate for evaluating the clinical prognosis of patients with PAH. In the clinical analysis to predict the prognosis of patients with PAH by echo parameters of cardiac function, we found that single ventricle diastolic dysfunction score, especially for LV diastolic dysfunction score (AUC_LVDD_  = 0.823, P<0.0001; AUC_RVDD_  = 0.737, P = 0.0002) had more accurate and significant value for predicting the clinical prognosis of patients with PAH than most single echo parameters such as E_m_, E_m_/A_m_, S_e_′, E_t_, A_t_, e_t_′, E_t_/A_t_, E_t_/e_t_′, DT_t_, TAPSE, Tei and FAC. Further analysis showed that bi-ventricular diastolic function scores (LVDD + RVDD scores) were more predictive than single ventricular diastolic dysfunction scores (LVDD score or RVDD score) (Table 5, Fig. 6). Moreover, the predictive data from bi-ventricular diastolic function scores were close to the predictive values of PVR_RHC_ (AUC_LVDD + RVDD_  = 0.825 vs. AUC_PVR_  = 0.838, both P<0.0001).

**Table 5:** Results of ROC curve analysis comparing different parameters of RHC, different echocardiographic parameters of left/right ventricular function, LVDD score, RVDD score and (LVDD + RVDD) scores for their accuracy in predicting unfavorable outcomes after the last follow-up.

Parameter	AUC	95%CI	*P*	Cutoff	Youden index	Sensitivity (%)	Specificity (%)
SPAP	0.780	0.652–0.878	<0.0001	69 mm Hg	0.4833	65.0	83.3
DPAP	0.790	0.663–0.886	<0.0001	37 mm Hg	0.5083	67.5	83.3
MPAP	0.807	0.682–0.899	<0.0001	51 mm Hg	0.4778	70.0	77.8
CO^#^	0.649	0.512–0.769	0.0812	2.9 L/min	0.2889	90.0	38.9
PVR	0.838	0.717–0.912	<0.0001	12.9 Wood Units	0.6028	82.5	77.8
E_m_	0.662	0.526–0.781	0.0492	85.18 cm/s	0.344	89.0	44.4
E_m_/A_m_	0.726	0.593–0.835	0.0018	1.29	0.3917	72.5	66.7
e_m_′	0.804	0.679–0.897	<0.0001	8 cm/s	0.5194	57.5	94.4
E_m_/e_m_′	0.806	0.681–0.898	<0.0001	7.14	0.5889	70.0	88.9
Septal e_s_′	0.650	0.513–0.771	0.0306	8 cm/s	0.2944	35.0	94.4
DT_m_	0.794	0.668–0.889	<0.0001	168 msec	0.5528	77.5	77.8
E_t_	0.634	0.497–0.757	0.1007	66 cm/s	0.3056	75.0	55.6
A_t_	0.622	0.485–0.746	0.123	64 cm/s	0.1833	35.0	83.3
E_t_/A_t_	0.644	0.508–0.766	0.0793	1.3654	0.3306	77.5	55.6
e_t_′	0.570	0.433–0.699	0.3725	14 cm/s	0.175	17.5	100
E_t_/e_t_′	0.626	0.489–0.750	0.0975	4.4	0.2944	35.0	94.4
DT_t_	0.556	0.419–0.686	0.5146	161 msec	0.1889	80.0	38.9
TAPSE	0.743	0.611–0.849	0.0004	13 mm	0.3806	82.5	55.6
Tei	0.553	0.416–0.684	0.515	0.5	0.175	17.5	100
FAC	0.605	0.468–0.731	0.2053	0.3	0.2194	77.5	44.4
LVDD score	0.823	0.700–0.911	<0.0001	2	0.5639	67.5	88.9
RVDD score	0.737	0.605–0.844	0.0002	2	0.3833	55.0	83.3
(LVDD + RVDD) scores	0.825	0.703–0.912	<0.0001	4	0.55	55.0	100

CO^#^: Cardiac output in CHD-PAH was measured by Fick's method; E_m_: Mitral valve peak E velocity; A_m_: Mitral valve peak A velocity; e_m_′: Lateral e velocity of mitral annulus by DTI; DT_m_: Deceleration time of mitral valve peak E; E_t_: Tricuspid valve peak E velocity; A_t_: Tricuspid valve peak A velocity; e_t_′: Lateral e velocity of tricuspid annulus by DTI; DT_t_: Deceleration time of tricuspid valve peak E.

## Discussion

### Cardiac function

Clinicians have paid increasing attention to the impairment of LV diastolic function in patients with PAH. However, there is no direct measure of LV stiffness and relaxation function and no accepted comprehensive evaluation index of LV diastolic function. The present studies [23]-[25] have assessed LV diastolic function of patients with PAH through single or multiple echo parameters (E_m_, A_m_, E_m_/A_m_, DT_m_, e_m_′, E_m_/e_m_′, a_m_′, S_m_′). Each parameter emphasizes a different aspect of LV diastolic function in PAH. It is well known that no single echo parameter is capable of accurately discriminating between normal and various levels of severity of LV diastolic dysfunction, because most indicators are influenced by multiple factors, including myocardial compliance and stiffness, LV systolic function and cardiac rhythm [26]. Our study integrates multiple parameters to modify LV diastolic function and quantifies the grading scheme (normal, grade I, grade II and grade III) into LV diastolic function scores 1, 2, 3 and 4, in order to analyze LV diastolic function intuitively and comprehensively. Our study shows a statistically significant correlation between LVDD score and 6MWD. Moreover, in the ROC curve analysis, the area under the ROC curve for LVDD score is similar to that for PVR by RHC. Using the LVDD score to evaluate the clinical prognosis of patients with PAH can overcome the lack of an individual indicator with sufficient reference value, making the data more comprehensive and robust enough to encompass the actual clinical situation of patients with PAH.

Although echo is a recommended screening test for pulmonary hypertension, its limited sensitivity and only moderate correlation with invasively-determined SPAP are notable limitations [8], [27], [28]. In addition, the short-term variability of RVSP measurements is unknown; thus, RVSP elevation requires confirmation with RHC in an individual patient.

In recent years, in order to assess right heart function in patients with PAH and to further predict the clinical prognosis of patients with PAH, TAPSE [1], [2], FAC [29], [30], and Tei index [4], [5], measured by 2D-echo, have been studied [31]. These parameters derived from 2D-echo for RV functional assessment have been proposed, but all have inherent strengths and weaknesses. TAPSE can reflect longitudinal RV movement rather than short-axis movement [1]. TAPSE alone may not be a good parameter to assess pre- and afterload variations for the RV but simply reflects impaired RV function [2]. FAC has the advantage that it quantifies shortening of both the septum and the RV free wall and is shown to correlate well with global RV function. However, the tracing of the RV endocardial area by 2D-echo is difficult because of the limited range of the echocardiographic window and the trabecular endocardial surface, thereby limiting the use of FAC in clinical practice [30]. Ogihara *et al*. demonstrated that improvement in Tei index but not TAPSE and FAC correlated with improvements of PVR during the clinical course in patients with PAH. However, Tei index did not significantly correlate with 6MWD at follow up [4]. TAPSE, FAC and Tei index can reflect part of the right heart function to some degree, but not the entire right heart function. Single parameter (TAPSE, FAC and Tei index) may be not very comprehensive and accurate to reflect the whole right heart function. In order to assess the entire right heart function, we integrated multiple parameters and quantified the different degree of RV diastolic function into scores of 1, 2, 3, and 4. Bivariate correlation analysis revealed a significant correlation between RVDD score and 6MWD. It is well known that RV and LV operate as a entirety, so the diastolic function of one ventricle may influence that of the other, which is well recognized as ventricular interdependence. In the quantitative analysis of ventricular function, our study analyzed LV and RV diastolic function as a whole by adding the LVDD score to the RVDD score. We created a new quantitative index, bi-ventricular diastolic function scores, which shows a greater significant correlation with 6MWD, and also has a significant predictive value for the prognosis of patients with PAH. A major advantage of LVDD/RVDD score is its ability to offer comprehensive and quantitative grades of ventricular diastolic function without the limitations inherent to other 2D-echo derived parameters of ventricular diastolic function. Quantitative grades of ventricular diastolic function will aid in clinical decision making, as echo assessments of cardiac function are routinely used to guide the escalation of PAH therapy.

In this study, we created new indices of cardiac diastolic functional classification (LVDD score, RVDD score and LVDD + RVDD scores) to comprehensively assess the clinical prognosis of patients with PAH. This is the first study to use quantitative grades of ventricular diastolic function to evaluate clinical outcomes of patients with PAH, and the results show significant correlations between the quantitative scores of ventricular diastolic function score (LVDD score, RVDD score and LVDD + RVDD scores) and 6MWD. In our study, as well as others [3], [6], [7], although 2D-echo assessment of LV or RV systolic and diastolic function based on E_m_ [7], TAPSE [3], FAC [29], [30] and Tei index [6], can predict clinical outcomes in patients with PAH, the predictive value of those parameters is only available for patients who already have moderate or severe impairment of cardiac function. The newly-developed quantitative and comprehensive indices (LVDD score, RVDD score and LVDD + RVDD scores) are more widely suitable for different degrees of left/right cardiac impairment in patients with PAH. However, 2D-echo can only track motion occurring within the imaging plane, thus it is intrinsically limited by its 2D nature. Three-dimensional echocardiography (3D-echo) can overcome 2D-echo limitations by neglecting geometric assumptions and using multiple images to draw the outline of RV geometry and reconstruct the RV chamber [32], [33]. And 3D-echo exhibits better reproducibility and reliability in the assessment of RV volumes and function compared to a gold standard technique such as MRI [34]. Therefore, this findings needs further investigation by 3D-echo.

### Limitations

Several study limitations should be pointed out. First, this study had no control group. Second, our results should be confirmed in a larger study population. Our study included heterogeneous causes of WSPH-I, with a variety of treatments and follow-up periods. In addition, because of the small sample population and too many risk factors, we could not perform subgroup analysis and uni- and multivariate logistic analysis. Third, in our study, as well as others [26], individual measurements failed to fall into predetermined grades whether in the algorithm of LVDD or RVDD score. In order to analyze LV or RV diastolic function intuitively and statistically, our study selected the grade which more parameters fit into as the object of study. Besides, bi-ventricular diastolic dysfunction was quantified by 2D-echo parameters. However, there were some missing data: (1) mitral diastolic blood flow velocity curve change of E/A before and after Valsalva test; (2) pulmonary vein blood flow peak velocity curve (S, D); (3) pulmonary vein blood flow reverse, blood flow velocity and duration (Ar). Finally, although 6MWD has been shown to have only modest validity as a surrogate endpoint for clinical events [35], our study was designed in 2008 when 6MWD was viewed as a surrogate endpoint for clinical events in patients with PAH.

## Conclusion

Single ventricular diastolic function score was superior to single parameters measured by 2D-echo for predicting clinical prognosis in patients with PAH, and bi-ventricular diastolic function scores were better than single ventricular diastolic function score for predicting clinical outcomes in patients with PAH. LVDD/RVDD/LVDD+RVDD score yielded a comprehensive quantitative assessment of LV/RV diastolic function that correlated moderately with clinical functional parameters and might be useful in the assessment of PAH.
